# Setting Up and Assessing a New Micro-Structured Waveguiding Fluorescent Architecture on Glass Entirely Elaborated by Sol–Gel Processing

**DOI:** 10.3390/ma15030979

**Published:** 2022-01-27

**Authors:** Morgane Bonnel, Ibtihel Marzouk, David Riassetto, Alain Morand, Davide Bucci, Michel Langlet

**Affiliations:** 1 LMGP, Grenoble INP, CNRS, University Grenoble Alpes, 38000 Grenoble, France; morgane.bonnel@live.fr (M.B.); ibtihel.marzouk@grenoble-inp.fr (I.M.); David.Riassetto@grenoble-inp.fr (D.R.); 2 IMEP-LAHC, Grenoble INP, CNRS, University Grenoble Alpes, 38000 Grenoble, France; alain.morand@grenoble-inp.fr (A.M.); davide.bucci@phelma.grenoble-inp.fr (D.B.)

**Keywords:** sol–gel process, channel waveguide, diffraction grating, fluorescence

## Abstract

Channel waveguides with diffraction gratings at their input and output for light injection and extraction, respectively, are extensively exploited for optical and photonic applications. In this paper, we report for the first time on such an architecture on glass entirely elaborated by sol–gel processing using a titanium-oxide-based photoresist that can be imprinted through a single photolithography step. This work is more particularly focused on a fluorescent architecture including channel waveguides doped with a ruthenium-complex fluorophore (tris(4,7-diphenyl-1,10-phenanthroline)ruthenium(II), Rudpp). The study demonstrates that this original sol–gel micro-structured architecture is well adapted to efficient channel waveguide/diffraction grating coupling and propagation of the fluorescence excitation and emission signals in the core of the channel waveguide. It demonstrates, in particular, a relatively large tolerance of several degrees in the angular injection fiber positioning and an important axial and vertical fiber spatial positioning tolerance of more than 100 µm at the Rudpp emission wavelength. The measurements also indicate that, in the conditions tested in this work, a Rudpp concentration of around 0.1 mM and a channel waveguide length of 2 to 5 mm offer the best trade-off in terms of excitation signal propagation and emission signal detection. This work constitutes a promising preliminary step toward the integration of our architecture into a microfluidic platform for fluorescence measurement in a liquid medium and waveguiding configuration.

## 1. Introduction

In past years, miniaturized components in the form of integrated planar or channel waveguides have largely been exploited for optical and photonic applications. These structures rely on the well-known principle of light confinement. In a planar configuration, this confinement is achieved through a suitable control of the waveguide core thickness as well as a suitable mismatch between its refractive index and those of the substrate and superstrate. This arrangement allows a vertical confinement of light and its propagation mainly through the high-refractive-index core by total internal reflection. In a channel waveguide having a finite width, the optical power field is confined in both directions. Over the two past decades, such integrated waveguides have been extensively studied as optical sensors to probe biomolecules or monitor physical–chemical parameters in biological and biomedical applications. In planar or channel waveguide configurations, the presence of a target analyte in the surrounding medium induces changes in optical properties (absorption coefficient, refractive index, fluorescence intensity, plasmon resonance, etc.) at the waveguide surface. This in turn modifies the signal propagating along the waveguide core. These modifications can then be measured with traditional sensitive photon detection techniques such as photomultiplier tubes and charge-coupled devices. Derived optical biosensors are integrated into microfluidic platforms adapted to measurements in a liquid medium, which results in the fabrication of miniaturized and portable sensors. Table 1 illustrates several examples of such biosensors cited in the literature [1,2,3,4,5,6,7,8,9,10,11,12,13], and many other examples can be found in recent reviews [14,15,16].

These studies illustrate the widespread range of probed analytes and sensing principles that can be considered using optical waveguides intended for biological and biomedical applications. They also highlight the fact that, because of light confinement in the core of the waveguide, the increased light–analyte interaction results in enhanced sensitivity and fast response, which offers an optimal solution for real-time and on-site detection. In this context, the sol–gel process has been reported to be a suitable route for the elaboration of waveguide sensors (see a recent review in [17]), and some papers report on the sol–gel processing of waveguide-based integrated biosensors [1,5,7]. These studies demonstrate that the sol–gel process provides an ideal trade-off between low cost, easy implementation and optical quality, as well as mechanical and chemical robustness, and in particular, it can be conveniently implemented without employing any step requiring a clean room.

The main issue in the exploitation of planar or channel waveguides is the achievement of efficient light coupling with the waveguide core, and this issue appears all the more challenging when considering device integration into a microfluidic platform for light measurements in a liquid medium. Coupling is often performed by end-fire butting using an optical fiber, but this method is rather demanding because it necessitates a rigorous end-facet polishing of the waveguide, and in the case of thin waveguides it requires a critical fiber–waveguide alignment tolerance. Several approaches have been considered to solve these problems, e.g., micro-lensed fibers [18] or different variants of taper couplers [19,20,21]. Another alternative relies on light coupling with diffraction gratings [22,23]. This type of coupling appears very promising since diffraction gratings exhibit relaxed positioning tolerances, do not require waveguide facet polishing, and are fully compatible with integration into miniaturized devices. Furthermore, this type of excitation is less noisy than the butt coupling case. In the latter, leaky modes and a radiated field can be superposed on the guided mode at the waveguide output. With diffraction gratings, this noisy light is essentially transmitted vertically to the substrate plane. The contrast at the end of the waveguide is increased. The use of diffracting couplers requires a periodic structure composed of fine patterns whose period should ideally be of the order of the considered wavelength. This coupling method has already been exploited in some planar- or channel-waveguide-based optical biosensors [24,25,26]. However, the method has only been the object of recent studies in this field and its potential has so far not been sufficiently examined. Furthermore, despite the well-recognized potential of sol–gel-derived integrated devices, only very rare papers report on the sol–gel fabrication of integrated waveguides endowed with grating couplers. Enami reported on such a sol–gel-derived grating elaborated with a nanoimprinting method, and he mentioned the difficulty of fabricating a grating on a large area [27]. Accordingly, a grating of only a few hundred microns in length was obtained. This lack of studies devoted to sol–gel methods and their derived performances presumably illustrates that the presently available knowhow in sol–gel processing is not yet sufficiently well adapted. These features introduce the objectives and originality of the work described in the present paper.

We recently presented an original sol–gel procedure that can strongly enhance the necessary knowhow [28]. In this work, we took advantage of a TiO_2_-based sol–gel photoresist that can be imprinted through a single insolation/development photolithography step. A specific procedure based on this photoresist and leading to micro-structured architectures composed of diffraction gratings imprinted on channel waveguides was thus proposed. We particularly demonstrated the possibility of elaborating gratings with large areas with lengths of 5 mm or more. The channel waveguide was also doped with a model fluorophore commonly used for dissolved oxygen (DO) sensing in the real-time control and monitoring of cell cultures in the biomedical field. This fluorophore (tris(4,7-diphenyl-1,10-phenanthroline)ruthenium(II), Rudpp) exhibits large excitation and emission bands around 460 and 610 nm, respectively. In the presence of oxygen, the fluorescence emission of Rudpp is selectively quenched. Thus, the emission intensity or the excited-state lifetime decreases with increasing molecular oxygen concentration, which allows sensitive DO detection in waveguiding [1] or other [29,30,31] configurations. However, studies devoted to Rudpp generally rely on its encapsulation in an organic polymer or silica-based sol–gel host matrix [1,29,30] whose weak refractive index hardly fulfills light-guiding requirements, i.e., a suitable index mismatch with the substrate. The high index of a TiO_2_-based matrix allows these requirements to be fulfilled, and such a sol–gel derived matrix has already been proposed for the encapsulation of a Ru-complex fluorophore [31], but the micro-structuration of a TiO_2_-based sol–gel matrix doped with Rudpp has never been studied for the fabrication of integrated channel waveguides. In our previous work, we demonstrated that the proposed TiO_2_-based photoresist is compatible with Rudpp encapsulation and oxygen sensitivity, showing that it is compatible with the diffusion of molecular oxygen in its thickness, for contacting Rudpp and activating its photoresponse. In that work, the micro-structured architectures were elaborated on silicon wafers, which required a low-refractive-index cladding sub-layer intended to suppress any optical interaction between the waveguide and the substrate optical absorption and reflection that would perturb the light propagation. However, we also observed that some heterogeneous defects of the sol–gel cladding sub-layer induced important optical losses, which hindered an accurate assessment of the potential of our waveguiding architecture. Such a feature would require an optimization of the cladding sub-layer. Furthermore, preliminary deposition of a cladding sub-layer lengthens and complicates the whole experimental procedure.

In the present paper, we describe, for the first time, investigations performed on a micro-structured waveguiding fluorescent architecture elaborated by sol–gel processing on a transparent and low-refractive-index glass substrate that does not require the use of a cladding sub-layer. This work constitutes an essential first step toward the ultimate integration of our architecture into a microfluidic platform for fluorescence measurements in a liquid medium and a waveguiding configuration. Indeed, before any integration, it is crucial to define the best set-up conditions and to assess the ability of our architecture to efficiently couple and propagate the fluorescence excitation and emission signals. Hereafter, we firstly present the whole experimental procedure and the opto-geometrical properties of the channel waveguides and diffraction gratings constituting the obtained architecture on glass. We then focus on light coupling assessments and optimization using this architecture. In the final part of the paper, the study is completed by performing fluorescence measurements in the waveguide configuration, finally showing that our micro-structured architecture on glass is well adapted to efficient channel waveguide/diffraction grating coupling and propagation of the excitation and emission signals in the core of the channel waveguide. To summarize, we present here an original work dealing with a fluorescent channel waveguide/diffracting coupler architecture taking advantage of a Ti-based photoresist and entirely elaborated by sol–gel processing, which has never been described before in the literature. Compared to our previous work [28], the real novelty is that we are now able to propose a simplified and optimized procedure on glass leading to a well-controlled architecture that allows reliable assessments, as described in this paper.

## 2. Materials and Methods

### 2.1. Sol–Gel Processing

A transparent and high-refractive-index (n~1.85 and n~1.75 at the Rudpp excitation (460 nm) and emission wavelength (610 nm), respectively [28]) TiO_2_-based guiding layer was deposited using a low-temperature sol–gel procedure that has been fully detailed in a previous paper [32]. Low-temperature processing constitutes an essential feature for preserving the integrity and photo-activity of the Rudpp, which is then encapsulated in the guiding layer. Briefly, a sol was prepared from tetraisopropylorthotitanate (TIPT) and benzoylacetone (BzAc) diluted in a methanol/butanol/deionized water/hydrochloric acid mixture. Both chemicals were purchased from Sigma-Aldrich (Saint-Quentin-Fallavier, France). The BzAc/TIPT molar ratio and TIPT concentration were fixed at 0.6 and 0.5 M, respectively. To deposit waveguiding layers doped with the fluorophore, a stock solution with a Rudpp concentration of 12.5 mM was prepared in absolute ethanol, and various volumes of this solution were added to the Ti-BzAc sol to vary the final Rudpp concentration between 0.2 and 2 mM. Rudpp was purchased from Alfa Aesar (Kandel, Germany). The sol was then deposited by spin coating at 3000 rpm on 2.5 × 2.5 cm^2^ soda-lime glass substrates (purchased from Thermo Fisher Scientific, Courtaboeuf, France, refractive index of ~1.52 in the range of wavelengths studied in this work). The obtained liquid films were subsequently dried at room temperature and then heat-treated at 110 °C for 10 min, leading to doped or undoped Ti-BzAc xerogel thin films. In this sol–gel formulation, the obtained Ti-BzAc complex plays a key role in the elaboration of a micro-structured architecture. On the one hand, such a complexation reduces the sol–gel reactivity of TIPT, so that Ti-BzAc xerogel films deposited at room temperature and heat-treated at 110 °C are chemically unstable and can easily be leached through simple washing with alcohols. On the other hand, BzAc is a photosensitive reagent that undergoes partial photolytic decomposition when exposed to UVA light. This decomposition leads to alcohol-insoluble species (carbonates, carboxylates) that promote the chemical stabilization of the Ti-BzAc xerogel film. These features introduce the principle of the TiO_2_-based sol–gel photoresist developed in our group [32]. This acts as a negative resist where, after selective insolation and appropriate washing, the areas exposed to UVA light remain intact while non-exposed areas are totally removed from the substrate. The photoresist can therefore be imprinted through a single photolithography step, which has been exploited in this work to elaborate channel waveguide/diffraction grating architectures.

### 2.2. Micro-Structured Architecture Elaboration

One of our final objectives is to propose a micro-structured architecture including Rudpp-doped channel waveguides endowed with diffracting couplers at their input and output. In such a configuration, the waveguide will propagate the excitation and emission signals, thus implicating a large quantity of fluorophores that should guarantee the relevant fluorescence intensity, while the diffraction couplers will provide efficient injection of the excitation signal into the waveguide core and efficient extraction of the emission signal toward a photodetector. In the preliminary studies detailed in this paper, we simplified the experimental procedure, and a diffraction grating was imprinted only at one extremity of the waveguide. This simplified architecture was sufficient to couple excitation light by means of the grating and to allow the guided light emitted from the fluorophore to be collected. A two-step deposition/insolation lithographic procedure was implemented. In the first step, a Ti-BzAc xerogel film doped with Rudpp, deposited by spin coating and heat-treated at 110 °C, was insolated at 365 nm using a commercial device (UV-KUB from KLOE, Saint-Mathieu-de-Tréviers, France) in contact mode through a chromium mask. This mask was composed of linear UVA transparent stripes of 50 µm width covering the whole length (2.5 cm) of the insolated sample. A post-insolation heat treatment was then performed at 110 °C for 8 min to enhance the photo-induced solubility contrasts between insolated and non-insolated areas. After that, the derived patterns were developed in absolute ethanol, rinsed in deionized water to stop the development, and then gently dried with a nitrogen spray. This first step led to the elaboration of the channel waveguides. The same procedure was also used to elaborate planar waveguides, except that insolation was performed without the chromium mask, and no final ethanol development was necessary. The samples were finally heat-treated at 110 °C for two hours to enhance the waveguide chemical stability before the subsequent deposition of a new sol–gel thin film. The same procedure (deposition/heat-treatment at 110 °C/insolation through a chromium mask/post-treatment at 110 °C) was then repeated a second time and the sample was once again washed in ethanol to develop the diffraction grating (not doped with Rudpp) on the waveguide surfaces. As explained in the Introduction, the period of the diffraction grating should ideally be of the order of the wavelength of interest (around 460 and 610 nm in this case). However, owing to diffraction limits, well-resolved patterns of a width smaller than 1 µm could hardly be obtained under our conditions using traditional photolithography with a chromium mask. Thus, for this second lithographic step, we used a chromium mask composed of linear UVA transparent stripes with a width/pitch of 1 µm/2 µm located on a 0.5 × 1 cm^2^ area of the mask. The linear transparent stripes were positioned perpendicularly to the channel waveguides at one of their extremities.

### 2.3. Characterization

The opto-geometrical properties (profile, periodicity, height, and width) of the channel waveguides and diffraction grating constituting the micro-structured architecture were quantified by atomic force microscopy (AFM) with a Bruker Dimension Icon (Camarillo, CA, USA) device operated in tapping mode using a ScanAsyst-Air triangular geometry tip mounted on a silicon nitride cantilever. Their large-scale uniformity was evaluated by optical microscopy using a Leica apparatus. Light coupling between the channel waveguides and the diffraction grating was investigated using Thorlabs (Newtown, CT, USA) LP450-SF15 and S1FC635PM laser sources emitting at 450 and 635 nm, respectively. These wavelengths match the excitation and emission spectra of Rudpp fairly well (large bands around 460 and 610 nm, respectively). The signal emitted by the laser sources was injected into the sample to be characterized using a Thorlabs S405-XP single mode fiber with a 4 µm core diameter and a numerical aperture (NA) of 0.12, and a dedicated set-up allowing precise angular and XYZ positioning of the fiber was employed.

**Figure 1:**
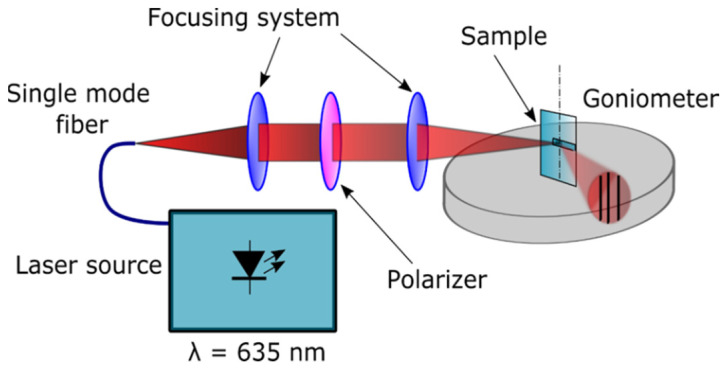
Schematic illustration of the modified M-lines bench.

Two sets of experiments were performed to assess the light coupling of the Rudpp emission signal using the 635 nm source. We firstly exploited a modified M-lines method. This method generally involves light coupling and decoupling with a planar or channel waveguide using a prism pressed onto the waveguide surface [33]. It can be used, for instance, to determine the effective index of the guided modes and corresponding guided-mode orders at a given wavelength, which enables the derivation of the guide thickness and refractive index. In this study, the method was adapted to light coupling and decoupling through the diffraction grating imprinted on a planar waveguide, as illustrated in Figure 1. A conical polarized light beam covering a wide range of angular incidences is reflected by the diffraction grating and subsequently illuminates a screen. However, this beam is not totally reflected since the diffraction grating is expected to induce light coupling in the waveguide core for a finite number of incidence angles. Black lines or missing lines (for M-lines) are thus observed on the illuminated screen, and their angular position enables various discrete values of diffraction angles leading to light coupling in the waveguide to be deduced. For that purpose, a scope equipped with a crosshair is manually aligned on each black line. A goniometer allows the angles to be obtained, measured with respect to the axis perpendicular to the sample surface and determined by means of a self-collimation technique. This manual procedure does not provide a recording of the line spectrum, but it is very flexible as the sample can be freely oriented with respect to the excitation beam.

**Figure 2:**
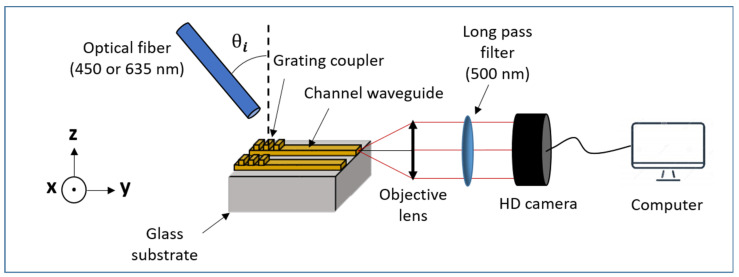
Schematic illustration of the bench used for light measurements in waveguide configuration.

In subsequent experiments, the 635 nm signal was injected into the core of the channel waveguides via the diffraction grating, and light propagating in the waveguides was collected from their output facet without the use of a diffracting coupler, as illustrated in Figure 2. To this end, the emerging signal was first focused on a Solinocam H2D2 camera through a Mitutoyo objective lens. The obtained images were then acquired through a FireWire card and analyzed with the LabVIEW software (National Instruments Corp., Austin, TX, USA), and derived pixel matrices were finally integrated onto the whole studied waveguide section and converted into light intensity units using the MATLAB software (MathWork Inc., Natick, MA, USA. These experiments enabled the angular and spatial positioning tolerance of the injection fiber to be assessed in relation to channel waveguide/diffraction grating coupling efficiency. Such assessments required a rigorous polishing of the output waveguide facet in order to optimize light extraction. To this end, the waveguide facet and supporting glass substrate section were firstly cut to a 200 µm depth using a high-precision saw from DISCO HI-TEC EUROPE (RBT6219/granulometry of 4000, Kirchheim, Germany) that allowed simultaneous cutting and fine polishing [34]. The 1 mm thick substrate was then cut more roughly to an additional depth of several hundred micrometers and finally cleaved. Sample cutting/polishing was performed in order to test the light propagation in waveguides of 2 or 5 mm in length. In further experiments, the same configuration was exploited, and the 450 nm source was used to assess light coupling at the Rudpp excitation wavelength through fluorescence emission measurements. The excitation signal emerging from the S405-XP fiber was injected into the Rudpp-doped waveguides via the diffraction grating, and the emission signal propagating in the waveguide core was collected from the polished output facet and analyzed as previously explained to derive the emerging light intensity. For comparison purposes, these measurements were performed on planar and channel waveguides. A reference fluorescence spectrum for a Rudpp-doped Ti-BzAc layer was also acquired in a non-guiding configuration. In this case, the excitation signal was injected perpendicularly to the doped layer, and the emission signal transmitted through the glass-supported layer was collected with a USB2000+ spectrophotometer from Ocean Optics (Orlando, FL, USA). For all these fluorescence measurements, a Thorlabs FEL0500 long-pass filter with a 500 nm cutoff wavelength was used to eliminate the excitation signal emerging from the waveguide core or transmitted through the glass-supported layer, thus allowing selective detection of the Rudpp emission. More detailed data on these optical characterizations are provided in the Results and Discussion section.

## 3. Results and Discussion

### 3.1. Opto-Geometrical Characterization

**Figure 3:**
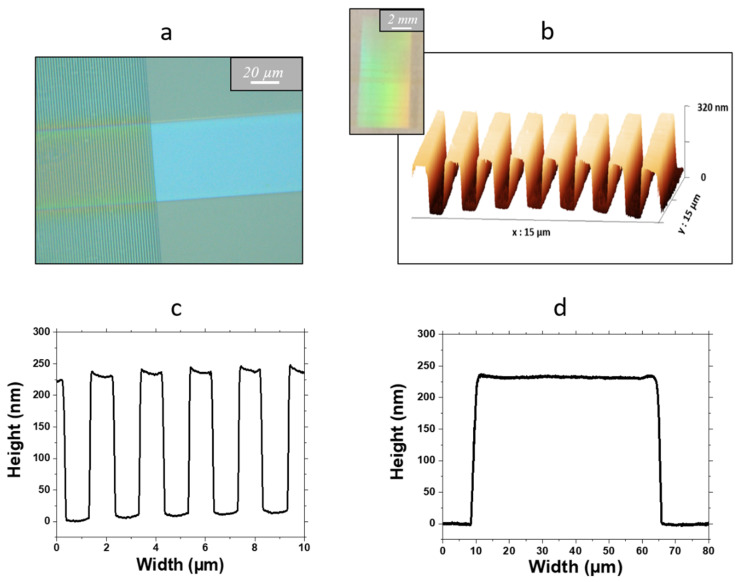
Top view optical micrograph of a diffraction grating imprinted at the input of a channel waveguide (**a**); 3D AFM image of a diffraction grating (**b**); typical AFM profile of a diffraction grating (**c**); typical AFM profile of a channel waveguide (**d**). In (**b**), the inset illustrates the whole area where a diffraction grating was imprinted.

The opto-geometrical properties of the channel waveguides and diffraction gratings constituting the micro-structured architecture on glass are illustrated in Figure 3. The micrograph in Figure 3a and the 3D AFM image in Figure 3b show that, for both components of this architecture, smooth and linear patterns were obtained, and a clean interface is depicted in Figure 3a between the bare channel waveguide and the area where the diffraction grating was imprinted (left part of the figure). The uniform diffraction effects illustrated in the inset of Figure 3b also show that the grating was homogeneously imprinted over the whole 0.5 × 1 cm^2^ mask area, where transparent stripes of 1 µm/2 µm width/pitch are present. The AFM profile in Figure 3c illustrates a typical diffraction grating whose width/pitch closely matches that of the mask and whose uniform height of 220 nm appears to be in close agreement with that previously obtained on silicon substrates [28]. In contrast, as illustrated in the AFM profile of Figure 3d, the channel waveguide width is slightly greater (55 µm) than the expected value of 50 µm, and its thickness of 230 nm is also slightly greater than that previously obtained on silicon. A longer post-insolation washing would probably have enabled more closely conforming channel waveguides to be obtained. However, these small variations do not significantly alter the behavior of the device. Indeed, we previously showed that such dimensions lead to single mode and multimode waveguides in their thickness and width, respectively, and that neither thickness nor width has a critical influence on light propagation and channel waveguide/diffraction grating coupling [28]. The AFM profiles of Figure 3c,d also depict photo-imprinted patterns that do not present vertical edges but rather exhibit a trapezoidal profile with edge slopes of around 45°. This feature, which was also observed for channel waveguides and diffraction gratings on silicon, is attributed to diffraction effects on the edges of the transparent stripes during insolation through the mask [32]. All these data globally demonstrate that our two-step deposition/insolation protocol initially developed on silicon substrates has been successfully extrapolated to the elaboration of a micro-structured architecture on glass with a similar quality.

### 3.2. Planar Waveguide/Diffraction Grating Coupling

Planar waveguide/diffraction grating coupling was preliminarily examined on the basis of theoretical considerations and optical simulation. Light propagating in a planar waveguide is described by two polarization modes, i.e., the transverse electric (TE) and transverse magnetic (TM) modes. For instance, if we consider a TE polarization (major electric field oriented parallel to the substrate plane) in the simplified case of a planar waveguide, the so-called dispersion relation for guided modes applies [35,36]:

(1) $$\frac{2\pi }{\lambda }d\sqrt{n_{c}^{2}-n_{\mathrm{eff}}^{2}}-\arctan\left(\sqrt{\frac{n_{\mathrm{eff}}^{2}-n_{\mathrm{sub}}^{2}}{n_{c}^{2}-n_{\mathrm{eff}}^{2}}}\right)-\arctan\left(\sqrt{\frac{n_{\mathrm{eff}}^{2}-n_{\sup}^{2}}{n_{c}^{2}-n_{\mathrm{eff}}^{2}}}\right)-m\pi =0$$

where *n*_c_, *n*_sub,_ and *n*_sup_ are the refractive indices of the waveguide core, the substrate, and the superstrate (or external medium), respectively, *d* is the waveguide thickness, and *λ* the wavelength. This equation can be numerically solved for a certain integer mode orders m, and when such a guided mode exists, it yields the corresponding effective index *n*_eff_. In our previous work, the waveguide effective indices were derived from Equation (1) using the thickness and refractive indices of Ti-BzAc layers determined from ellipsometric measurements [28]. For instance, in TE mode, we derived an *n*_eff_ value of 1.61 at 635 nm, which will be exploited in the following. The effective indices can then be used to assess light coupling at various wavelengths between the waveguides and diffraction gratings imprinted at their surfaces. In this configuration, light is injected into, or extracted from, the waveguide according to a finite number of diffraction angles θ_d_ by means of grating couplers having an appropriate period Λ. In order to achieve coupling with a guided mode of effective index n_eff_, the so-called grating law must be fulfilled [37]:

(2) $$n_{\sup}\sin\left(\theta_{d}\right)=n_{\mathrm{eff}}-\frac{q\lambda }{\Lambda }$$

where *q* is an integer that represents the diffracted coupling orders. Thus, Equation (2) enables θ*_d_* angles to be determined where light coupling toward the guided mode occurs from a plane-wave impinging on the grating or where guided light is radiated through the grating toward the external medium. However, this equation does not take into account the grating dimensionalities (height, width, profile, and length). Thus, optical simulation is necessary to derive more reliable insights into the light coupling efficiency.

**Figure 4:**
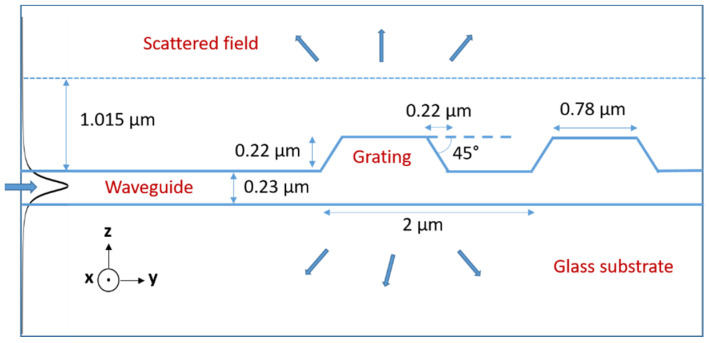
Schematic illustration of the model used for simulation.

**Figure 5:**
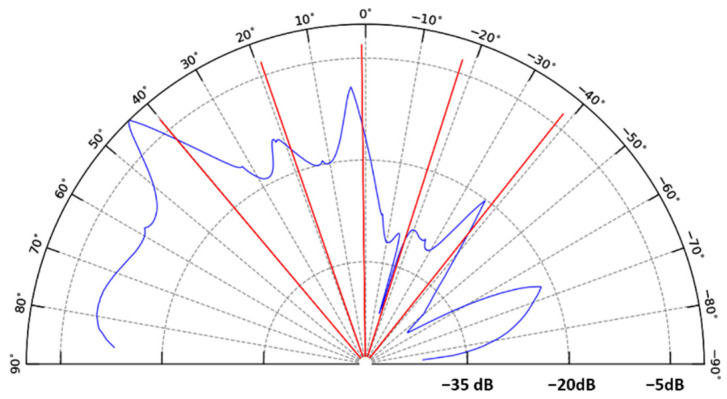
Intensity profile of light (*λ* = 635 nm, TE mode) extracted from a channel waveguide through a diffraction grating according to the geometry illustrated in Figure 4. The light intensity has been normalized according to the maximal value obtained for a diffraction angle θ*_d_* of around 44°. The red lines depict the theoretical diffraction angles deduced from the grating law (Equation (2)).

In this work, simulation was performed using the AFMM (aperiodic Fourier modal method), also known as RCWA (rigorous coupled-wave analysis) modified with PMLs (perfect matching layers). The method has been detailed in previous papers [38,39]. It involves a 2D simulation where electromagnetic fields and grating profiles are invariant in a transverse direction of the propagation. Hence, a planar waveguide and plane-wave excitation are used. In the experiment part, a lateral confinement waveguide is used. The method leads to the simultaneous determination of the diffraction angles θ*_d_* and their associated diffracted coupling orders q. For practical convenience, we considered a configuration where light propagating in the core of a channel waveguide was then extracted toward the external medium through the diffraction grating. However, owing to the principle of light reciprocity, conclusions derived from this configuration can rigorously be extrapolated to the reverse case, where the diffraction grating serves to inject light into the waveguide core. The method can be used to assess gratings with a square profile by taking into account their dimensionalities. For more complicated profiles, such as the trapezoidal profiles considered in this work, a spatial discretization of the sloping part (rising and falling edges) is necessary. This part was defined by a cascade of 21 layers with a width increment of 10 nm between each layer from top to bottom of the trapezoids (Y direction in Figure 4), and we used 163 spectral points in the vertical direction (Z direction in Figure 4). The period number of the grating was set to 50, i.e., a grating length of 100 µm, and the electric field was calculated along a line at a height of 1.015 µm above the waveguide, to calculate the angular radiation diagram. The other main parameters used in this simulation are schematized in Figure 4 according to previous opto-geometrical characterization, and an example of results derived from this simulation is shown in Figure 5 for a 635 nm signal in TE mode.

Figure 5 shows a series of peaks that depict light diffraction through the grating according to a finite number of extraction angles. The main peaks are located at around 44°, 22°, and 3° with respect to the vertical direction and are associated with coupling orders of three, four, and five, respectively. These peaks exhibit a certain broadness and are positioned at angle values slightly greater than those predicted by the grating law (also illustrated in Figure 5 for comparison purposes). This difference probably arises from the influence of the trapezoidal grating dimensionalities. In particular, the average thickness of the waveguide endowed with a grating is greater than that of a bare waveguide. A greater thickness yields a certain increase in the effective index of the actual waveguide, and this effect increases the diffracted angle, as seen in Equation (2). For simplification purposes, this feature has not been taken into account in this work. However, as discussed in the following, this simplification does not preclude drawing valuable information from the simulation. In addition, the observed peaks present different intensities that illustrate the efficiency of light coupling through the grating in relation to the extraction angle. Accordingly, the simulation illustrated in Figure 5 shows that the most efficient light coupling at 635 nm is achieved for an extraction (or injection) angle of 44° (vs. a theoretical value of 40° according to the grating law) and a coupling order of three.

Firstly, experimental studies were performed to evaluate the extent to which diffraction coupling follows trends predicted by the simulation. In these studies, the diffraction gratings were used to inject light into the waveguide core. This configuration is the opposite of that previously simulated. Nonetheless, as previously specified, both configurations can be compared owing to the principle of light reciprocity. We firstly employed the previously described modified M-lines method to characterize a diffraction grating imprinted on a planar waveguide. In accordance with the simulation illustrated in Figure 5, we considered light at 635 nm in the TE polarization. Three missing lines were detected for angles of around 39°, 18°, and 1° with respect to the vertical direction. As depicted in Figure 5, these angle values are in good agreement with those deduced from the grating law, and they appear slightly weaker than the optimal coupling angle values derived from simulation. The finest missing line was observed for a diffraction angle of 39°. According to the previously mentioned waveguide effective index of 1.61 at 635 nm, the grating law allowed a coupling order of q = 3 to be determined from this angle value, which closely matches the optimal coupling order deduced from simulation. The experimental data appear, therefore, to be in rather good agreement with theoretical and simulated data, and they provide the first evidence that light coupling occurs effectively between the planar waveguide and diffraction gratings.

### 3.3. Light Coupling and Fiber Positioning Tolerance Assessments

**Figure 6:**
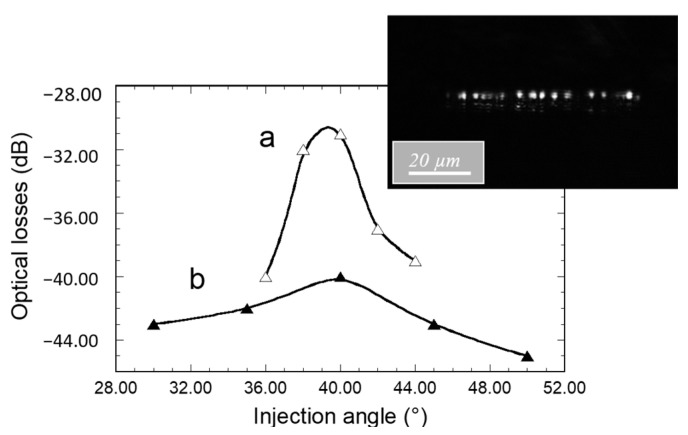
Influence of the injection fiber angular incidence on light emerging at the output facet of 55 µm width channel waveguides of 2 mm (**a**) and 5 mm (**b**) length after injection through diffraction gratings. The lines are drawn to guide the eye. In the inset, the optical micrograph illustrates a typical signal emerging from the 5 mm length waveguide section.

**Figure 7:**
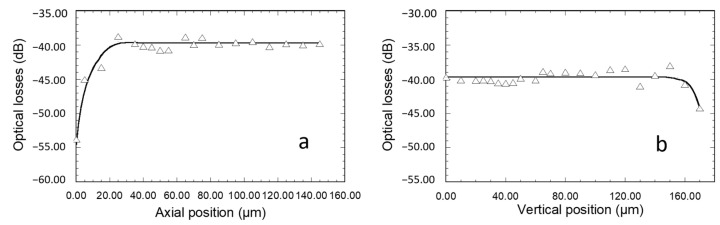
Influence of the injection fiber axial (**a**) and vertical (**b**) positions on the intensity of light emerging at the output facet of 55 µm width and 5 mm length channel waveguides after light injection through a diffraction grating at an incidence of 40°. The lines are drawn to guide the eye.

In a new set of experiments, light at 635 nm was injected through the diffraction grating into the core of 55 µm width channel waveguides, and the propagated signal emerging from the polished waveguide output section was analyzed using the MATLAB software, as previously explained. These experiments were intended to provide new evidence of efficient light coupling, and we paid particular attention to the injection fiber positioning tolerance versus the grating. This tolerance is of great relevance when considering our ultimate objectives, dealing with the integration of the micro-structured architecture into a microfluidic platform devoted to fluorescence measurements in a liquid medium. Indeed, in such a configuration, positioning of the fibers enabling efficient injection of the excitation signal and efficient collect of the emission signal through diffraction gratings can be rather tricky and particularly demanding with regard to fiber position tolerance. Figure 6 and Figure 7a,b illustrate how the angular (θ_i_ in Figure 2), axial (Y direction in Figure 2), and vertical (Z direction in Figure 2) positioning of the fiber influence the emerging signal intensity, respectively. First of all, it is important to note the correlation of experimental points in the curves of these figures, which illustrates well the high reproducibility and reliability of our measurements, i.e., the low measurement error. A typical signal emerging from the waveguide polished section is illustrated in the inset of Figure 6, which depicts the light spots associated with many guided modes propagating in the waveguide core. On the one hand, it confirms that, due to their low thickness (230 nm), the waveguides are single mode in the vertical direction. The spot diameters are obviously larger than the waveguide core thickness, owing to light diffraction at the output facet. On the other hand, the figure illustrates a multi-mode waveguide in its width direction, i.e., several spots distributed in the waveguide width that depict the result of the interference between multiple propagated modes. These features provide new evidence that diffraction gratings effectively enable light injection into channel waveguides, and that these particular ones allow an efficient guided mode confinement in the vertical and lateral directions. The influence of the light incidence was studied for channel waveguides of length 2 and 5 mm (Figure 6). To this end, the 4 µm core diameter injection fiber was centered on the waveguide width and positioned in contact with the diffraction grating and at a 60 µm distance from the interface between the grating and the bare waveguide. The data are given in terms of optical losses deduced from the emerging-to-injection light intensity ratio. For both studied waveguide lengths, Figure 6 shows that the angle dependence of the extracted light intensity follows an approximately Gaussian distribution with a rather large width at mid-height around an optimal injection angle of 39–40°. This optimal value is in very close agreement with that derived from previous M-lines analyses. According to this concordance, the slightly greater angle values deduced from simulation suggest that the latter can again be optimized, for instance by taking into account the influence of the grating on the waveguide effective index. Besides, the small divergence between experimental and simulated data probably arises from the difficulty of rigorously accounting for the overall characteristics of the grating (uniformity, reproducibility, etc.). However, these divergences should be relativized when considering that, owing to the broadness of peaks illustrated in simulation data of Figure 5, the angular dependence of diffraction coupling efficiency depicted by these peaks largely overlaps the experimentally deduced optimal coupling angles. Thus, simulation still provides a valuable guide to predicting experimental conditions. Furthermore, simulation and experimental data illustrate a tolerance of several degrees in the angular fiber positioning.

For an optimal injection angle of 39–40°, Figure 6 depicts optical losses of 30 and 40 dB for a waveguide length of 2 and 5 mm, respectively. We firstly infer that a great part of these losses is related to the light coupling efficiency through the grating. As mentioned in the Introduction, an optimized coupling efficiency requires a grating periodicity of the order of the considered wavelength in such a way that only one coupling order is involved, as shown by Equation (2). In our case, the simulation illustrated in Figure 5 shows that coupling through our gratings involves several orders, owing to their 2 µm periodicity. Thus, the coupling efficiency is strongly reduced and an important part of the light emerging from the injection fiber is lost by reflection at, or transmission through, the substrate. The losses illustrated in Figure 6 also arise from propagation in the waveguide cores. Since injection losses are inferred to be identical for the waveguides illustrated in this figure, optical loss differences between both waveguide lengths essentially depict such propagation losses. Considering the data illustrated in Figure 6 for an injection angle of 39–40°, these losses were roughly estimated to be around 3 dB/mm, i.e., around 6 dB and 15 dB for the 2 mm and 5 mm length channel waveguides, respectively. Such injection and propagation losses are obviously important, but in this work, our priority was not to optimize channel waveguide and diffraction grating performances but to propose a micro-structured architecture allowing the well-controlled and reproducible measurement of the propagated signal intensity. This is illustrated in the following for channel waveguides of 55 µm width and 5 mm length and for an injection angle of 40° with respect to the vertical direction since this angle shows a reasonable agreement between simulated and experimentally deduced values.

Figure 7a illustrates how the axial position of the fiber influences light emerging from the waveguide output section. The fiber, centered along the waveguide width at a few micrometers above the sample surface, was initially positioned in the vertical direction of the bare waveguide, just at the interface with the diffraction grating. No signal could be observed at the waveguide output facet, showing that light injection into the waveguide did not occur without the diffraction grating. In this case, the measured light and the corresponding optical losses of 55 dB were only related to ambient noise. Then, the fiber was progressively moved along the diffraction grating. Optical losses dropped to a value of around 45 dB after positioning the fiber in the vertical direction of the grating just at the interface with the bare waveguide, showing that light coupling had started to occur. Then, they underwent a further decrease to 40 dB when the fiber was gradually moved up to a distance of 20 µm from the interface with the bare waveguide. Finally, no loss evolution was detected with further fiber displacement up to a length of at least 120 µm, and optical losses of 40 +/−1 dB were measured. On the one hand, these data show that optimal light coupling with the channel waveguide requires a grating length of 20 µm (ten grating periods) or more. On the other hand, they show that when coupling is achieved far away from the bare channel waveguide (at least 120 µm), the eventual diffraction effects do not induce significant decoupling losses toward the external medium of the light propagating in the waveguide underneath the grating. It therefore allows an important degree of tolerance in the fiber axial positioning and the achievement of reproducible intensity measurements. A similar study was then performed to assess the tolerance in vertical positioning. The fiber was initially centered on the width of the waveguide and positioned in contact with the diffraction grating at 60 µm from the interface with the bare waveguide (in the middle of the plateau illustrated in Figure 7a), and it was then progressively moved vertically away from the grating. Figure 7b shows that reproducible losses of 40 +/−1 dB were measured for a fiber vertical displacement of around 150 µm, which illustrates once again an important fiber positioning tolerance allowing reproducible measurements. Then, optical losses started to increase with further vertical movement of the fiber. To summarize, the data in Figure 6 and Figure 7 illustrate large tolerances in the angular (several degrees) and spatial (more than 100 µm in axial and vertical directions) fiber positioning.

### 3.4. Fluorescence Measurements

**Figure 8:**
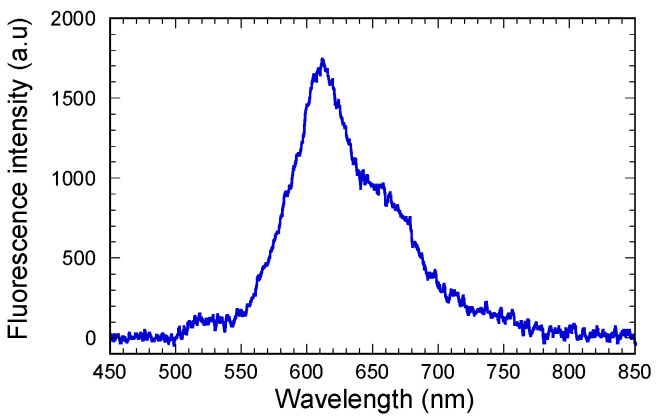
Fluorescence spectrum measured in transmission through a Rudpp-doped Ti-BzAc layer on glass under excitation at 450 nm. The layer was deposited from a sol with a Rudpp concentration of 1 mM.

Fluorescence measurements were performed under excitation at 450 nm to complete the assessments of light coupling through the diffraction gratings. A reference emission spectrum of a Ti-BzAc layer doped with the Rudpp fluorophore (1 mM in solution) was preliminarily collected by transmission through the glass-supported layer using a spectrophotometer and a long-pass filter with a 500 nm cutoff wavelength. As illustrated in Figure 8, the spectrum exhibits a large emission band extending between 550 and 750 nm, with a maximum at 610 nm. This figure particularly indicates that: (i) the 450 nm wavelength is well adapted to excite the Rudpp emission in our present experimental conditions, (ii) no significant signal is detected below 550 nm, showing that the long-pass filter used enabled the excitation signal to be totally eliminated, thus allowing selective detection of the Rudpp emission, and (iii) according to the important intensity measured at 635 nm, previous assessments performed at this wavelength provided reliable information on the waveguide/diffraction grating coupling of the Rudpp emission signal.

**Figure 9:**
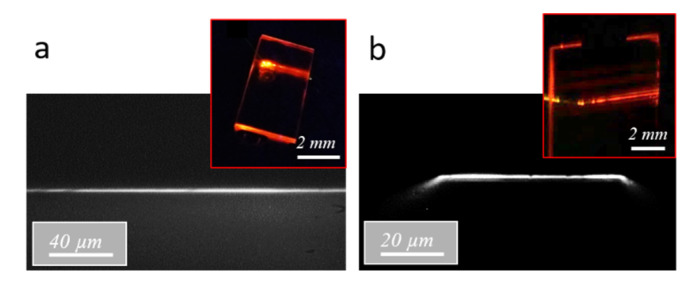
Optical micrographs of the signal emerging from the waveguide section after light injection through diffraction gratings at 450 nm in a Rudpp-doped planar waveguide of 2 mm length (**a**) and in a Rudpp-doped channel waveguide of 55 µm width and 5 mm length (**b**). The insets illustrate top-view pictures of the emitted signal propagating in the waveguides. For these pictures and their associated micrographs, a 500 nm cutoff wavelength filter was used to selectively visualize the fluorescence emission signal.

Fluorescence measurements were then performed in a guided light configuration on planar (i.e., 2D) and channel (i.e., 3D) waveguides endowed with diffraction gratings. The excitation signal was injected through the diffraction gratings in the core of the planar and channel waveguides at an incidence of 35° with respect to the vertical direction. This value, which corresponds to a coupling order of five at the 450 nm wavelength, was deduced from simulation (not illustrated here). The injection fiber was positioned in contact with the diffraction gratings at a 20 µm distance from the bare waveguides, and the emission signal was collected from the waveguide output polished facet through a 500 nm cutoff wavelength long-pass filter. As illustrated in the micrographs in Figure 9a,b, the emerging signal was well confined within the waveguide height, similarly to the signal emerging after light injection at 635 nm through the diffraction gratings (inset of Figure 6). For planar waveguides, no obvious lateral confinement was achieved, and the inset of Figure 9a shows that, after propagation, significant light can be detected over a width of about 400 µm at the waveguide output. In contrast, the micrograph in Figure 9b shows that the emission signal propagating in the channel waveguide was essentially confined in its 55 µm core width, although the inset of this figure also shows that a part of the signal seems to be radiated into the substrate underneath from either side of the waveguide. Figure 9b also depicts a slight signal inflexion at the edges of the channel waveguide output facet, which was not clearly explained in the present state. For both kinds of waveguides excited at 450 nm, a major difference from light injection at 635 nm was that the emerging signal was not composed of separated spots but appeared in the form of a continuous horizontal line. This line shows that, in this case, light does not propagate through discrete guided modes in a coherent way. The intensity tends to an average value due to the non-coherent fluorescence signal that is isotropically emitted in the space from Rudpp sources dispersed throughout the waveguide. This observation has an important practical consequence since it supposes, in particular, that only a part of the propagating fluorescence signal is actually confined within the waveguide cores, and that another important part is coupled with the substrate and the external medium.

**Figure 10:**
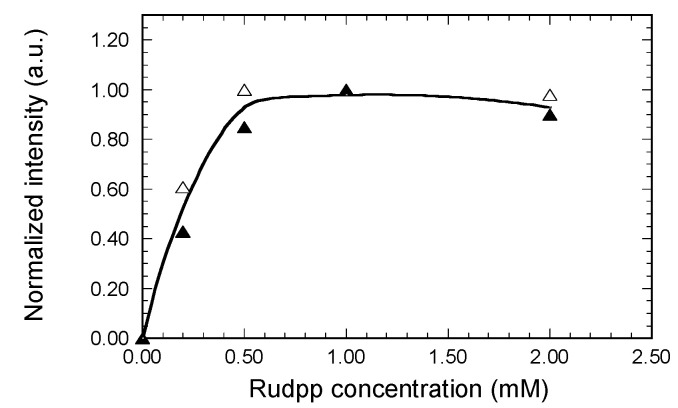
Influence of the Rudpp concentration in solution on the fluorescence intensity measured in guided configuration under excitation at 450 nm. The excitation signal was injected through diffraction gratings in the core of planar waveguides of 2 mm length (8) and channel waveguides of 55 µm width and 5 mm length (7), and the emission signal was collected from the output section of the waveguides. The line is drawn to guide the eye. For both kinds of waveguides, the fluorescence intensity has been normalized with respect to the maximal measured value.

Our experimental set-up was not adapted to direct intensity measurements of light emerging from the waveguide output facet using a spectrophotometer, especially in the case of a planar waveguide. This measurement should be possible with a micro-structured architecture where a diffraction grating imprinted on the waveguide output would allow extraction of the emitted light, which is one of our future objectives. In the present state, the Rudpp emission intensity was derived from the previously discussed MATLAB analysis, and the output signals analyzed in this way therefore accounted for the intensity integrated over the whole spectrum illustrated in Figure 8. However, fluorescence measurements required some adaptation of the MATLAB analysis. In previous experiments using light injection at 635 nm and propagation of guided modes, the emerging signal was analyzed from a pixel matrix of 1440 lines and 1920 columns. For fluorescence measurements, it was necessary to reduce the matrix dimensions in order to exclude the fluorescence extracted from the substrate and external medium and to selectively detect the emission signal emerging from the waveguide output. In the case of planar waveguides, this selective detection was achieved by reducing the number of pixel lines to 50, while the 1920 columns enabled the emission signal emerging from a 200 µm width section of the waveguides to be measured. For channel waveguides, the number of pixel columns was further reduced to 450 in order to also exclude fluorescence extracted from the edges of the 55 µm width waveguides. Derived fluorescence intensity measurements are illustrated in Figure 10 for various amounts of Rudpp fluorophore in the case of planar and channel waveguides of 2 mm and 5 mm length, respectively.

Interestingly, Figure 10 shows that both kinds of studied waveguides led to very similar trends, and in both illustrated curves the experimental points exhibit a good correlation, which once again illustrates the reproducibility and reliability of our measurements. In the planar waveguide, propagating light is expected to spread over a ~400 µm width (Figure 9a), compared with the 55 µm width in the channel waveguide (Figure 9b). Thus, the similar trends illustrated in Figure 10 show that the doping-level dependence of fluorescence intensity measured in the guiding configuration is not influenced by the propagation width. The fluorescence intensity logically increases with the Rudpp amount up to a concentration in solution of around 0.5 mM, after which it plateaus, with no further intensity increase or even a slight decrease above a Rudpp threshold concentration of 1 mM. The fact that fluorescence does not continuously increase with the fluorophore amount can firstly be attributed to parasitic phenomena intrinsically occurring when the concentration of fluorophore in the host matrix exceeds a certain threshold, e.g., concentration quenching or inner filter effects that reduce the fluorescence yield [40]. This phenomenon is also probably related to fluorescence measurements performed in the guided light configuration. Indeed, the excitation signal propagating along the waveguide cores is progressively absorbed by the Rudpp fluorophore. This means that, for a given Rudpp concentration, the excitation signal is totally absorbed after a threshold propagation length. Fluorophores located beyond this length are therefore not excited and no longer participate in the fluorescence emission, which can explain the intensity saturation depicted in Figure 10. Thus, these new data have two important practical consequences. On the one hand, they prove that coupling at the excitation wavelength is efficiently achieved through the diffraction gratings and allows detection of the Rudpp emission in a waveguiding configuration at the output of channel waveguides, thus providing new confirmation of the potential of our micro-structured architecture. On the other hand, they indicate that the best configuration should rely on a trade-off between the doping level of Rudpp (a concentration of around 0.1 mM in this study) and the length of channel waveguides (2 to 5 mm in this study) optimized in terms of excitation and emission signal propagation.

## 4. Conclusions

We have proposed and assessed a micro-structured fluorescent architecture on glass composed of channel waveguides and diffraction gratings entirely based on a sol–gel procedure. To the best of our knowledge, such a sol–gel-derived architecture has never been described before for optical or photonics applications. It particularly takes advantage of a transparent and high-refractive-index Ti-BzAc sol–gel photoresist that can be imprinted through a single photolithography step. In this study, simplified architectures including waveguides doped with Rudpp and endowed with a diffraction grating at only one extremity have been elaborated using a two-step deposition/insolation procedure. Theoretical simulation considerations based on the waveguide and grating opto-geometrical properties were first exploited to predict the best angular light coupling (around 35° and 40° at the 450 nm excitation wavelength and 610 nm emission wavelength of Rudpp, respectively) between both components of the sol–gel architecture. Experimental studies then confirmed these predictions in part, indicating a relatively large tolerance of several degrees in the angular fiber positioning and an important axial and vertical fiber spatial positioning tolerance of more than 100 µm at the Rudpp emission wavelength. Fluorescence measurements in the guided light configuration finally proved the compatibility of our architecture with light injection in the waveguide core at the Rudpp excitation wavelength and with the collection of the propagating Rudpp emission signal at the waveguide output facet. These measurements also indicated that, under the conditions tested in this work, a Rudpp concentration of around 0.1 mM and a channel waveguide length of 2 to 5 mm offer the best trade-off in terms of excitation signal propagation and emission signal detection. Despite the fact that our experimental conditions yielded some unwanted light coupling and propagation losses, this study showed that our sol–gel architecture is compatible with reproducible fluorescence emission measurements in a guided light configuration. This constitutes a promising preliminary step toward our subsequent objectives dealing with: (i) the elaboration of a more sophisticated architecture including diffraction gratings at the input and output of channel waveguides for injection and extraction of the fluorescence excitation and emission signals, respectively, (ii) integration into a microfluidic platform for fluorescence measurements in a liquid medium and a waveguiding configuration, and (iii) demonstration of the potential of our architecture on glass via the first dissolved oxygen sensing experiments based on the oxygen-driven extinction of Rudpp fluorescence.

## Figures and Tables

**Table 1:** Examples of optical waveguides intended for biological and biomedical applications.

Probed Analyte	Sensing Principle	Ref.
Dissolved oxygen	Fluorescence measurement	[1]
Dissolved carbon dioxide	Absorptiometry	[2]
Bovine serum albumin	Refractive index variation	[3]
pH	Fluorescence measurement	[4]
DNA	Labelled fluorescence measurement	[5]
	Plasmon resonance	[8]
Immunoglobulin G	Photocurrent measurement Labelled fluorescence measurement	[6] [7]
Glucose	Refractive index variation	[9]
Microcystin-LR	Labelled fluorescence measurement	[10]
Concanavalin A	Refractive index variation	[11]
C-reactive protein	Plasmon resonance	[12]
15-hydroxyvitamin D	Plasmon resonance	[13]

## Data Availability

Not applicable.

## References

[1] Hajj-Hassan M., Gonzalez T., Ghafar-Zadeh E., Djeghelian H., Chodavarapu V., Andrews M., Therriault D. (2008). Direct-dispense polymeric waveguides platform for optical chemical sensors. Sensors.

[2] Mayr T., Abel T., Enko B., Borisov S., Konrad C., Köstler S., Lamprecht B., Sax S., List E.J.W., Klimant I. (2009). A planar waveguide optical sensor employing simple light coupling. Analyst.

[3] Yan R., Mestas S.P., Yuan G., Dandy D.S., Lear K.L. (2009). Label-free silicon photonic biosensor system with integrated detector array. Lab Chip.

[4] Ram R.J., Lee K. Optical waveguides for microfluidic integration. Proceedings of the 2009 IEEE LEOS Annual Meeting Conference Proceedings.

[5] Bedu M., Sagarzazu G., Gacoin T., Audebert P., Weisbuch C., Martinelli L. (2010). Sol-gel planar waveguides for improved fluorescence microarrays. Thin Solid Film..

[6] Yan R., Lynn N.S., Kingry L.C., Yi Z., Slayden R.A., Dandy D.S., Lear K.L. (2011). Waveguide biosensor with integrated detector array for tuberculosis testing. Appl. Phys. Lett..

[7] Oubaha M., Gorin A., McDonagh C., Duffy B., Copperwhite R. (2015). Development of a multianalyte optical sol-gel biosensor for medical diagnostic. Sens. Actuators B Chem..

[8] Xing X., Liu W., Li T., Xing S., Fu X., Wu D., Liu D., Wang Z. (2016). A portable optical waveguide resonance light-scattering scanner for microarray detection. Analyst.

[9] Azuelos P., Girault P., Lorrain N., Poffo L., Guendouz M., Thual M., Lemaitre J., Pirasteh P., Hardy I., Charrier J. (2017). High sensitivity optical sensor based on polymer materials and using the Vernier effect. Opt. Express.

[10] Liu L., Zhou X., Wilkinson J.S., Hua P., Song B., Shi H. (2017). Integrated optical waveguide-based fluorescent immunosensor for fast and sensitive detection of microcystin-LR in lakes: Optimization and Analysis. Sci. Rep..

[11] Zeininger L., Weyandt E., Savagatrup S., Harvey K.S., Zhang Q., Zhao Y., Swager T.M. (2019). Waveguide-based chemo- and biosensors: Complex emulsion for the detection of caffeine and proteins. Lab Chip.

[12] Walter J.-G., Eilers A., Alwis L.S.M., Roth B.W., Bremer K. (2020). SPR biosensor based on polymer multi-mode optical waveguide and nanoparticle signal enhancement. Sensors.

[13] Walter J.-G., Alwis L.S.M., Roth B., Bremer K. (2020). All-optical planar waveguide-based biosensor chip designed for smartphone-assisted detection of vitamin D. Sensors.

[14] Benito-Pena E., Granda Valdes M., Glahn-Martinez B., Moreno-Bondi M.C. (2016). Fluorescence based fiber and planar waveguide biosensors: A review. Anal. Chim. Acta.

[15] Guo J., Yang C., Dai Q., Kong L. (2019). Soft and stretchable polymeric optical waveguide-based sensors for wearable and biomedical applications. Sensors.

[16] Steinegger A., Wolbeis O.S., Borisov S.M. (2020). Optical sensing and imaging of pH values: Spectroscopies, materials, and applications. Chem. Rev..

[17] Alberti S., Jagerska J. (2021). Sol-gel thin film processing for integrated waveguide sensors. Front. Mater..

[18] Le S.D., Delcourt E., Girault P., Guttierez-Arroyo A., Azuelos P., Lorrain N., Bodiou L., Poffo L., Goujon J.M., Dumeige Y. (2016). Study of optimized coupling based on micro-lensed fibers for fibers and photonic integrated circuits in the framework of telecommunication and sensing applications. Comm. Phys..

[19] Dai D., Tang Y., Bowers J.E. (2012). Mode conversion in tapered submicron silicon ridge optical waveguides. Opt. Exp..

[20] Cardenas J., Poitras C.B., Luke K., Luo L.W., Morton P.A., Lipson M. (2014). High coupling efficiency etched facet tapers in silicon waveguides. IEEE Photon. Technol. Lett..

[21] Liu J., Raja A.S., Pfeiffer M.H.P., Herkommer C., Guo H., Zervas M., Geiselmann M., Kippenberg T. (2018). Double inverse nanotapers for efficient light coupling to integrated photonic devices. Opt. Lett..

[22] Taillaert D., Van Laere F., Ayre M., Bogaerts W., Van Thourhout D., Bienstman P., Baets R. (2006). Grating couplers for coupling between optical fibers. Jpn. J. Appl. Phys..

[23] Demeter A., Ruschin S. (2016). Back-reflecting interferometric sensor based on grating coupler on a planar waveguide. J. Opt..

[24] Lambeck P.V. (2006). Integrated optical sensors for the chemical domain. Meas. Sci. Technol..

[25] Kuswandi B., Nuriman, Huskens J., Verboom W. (2007). Optical sensing systems for microfluidic devices: A review. Anal. Chim. Acta.

[26] Mukundan H., Anderson A.S., Grace W.K., Grace K.M., Hartman N., Martinez J.S., Swanson B.I. (2009). Waveguide-based biosensors for pathogen detection. Sensors.

[27] Enami Y. (2017). Fabricating 90 nm Resolution Structures in Sol-Gel Silica Optical Waveguides for Biosensor Applications. J. Sens..

[28] Bonnel M., Riassetto D., Morand A., Bucci D., Langlet M. (2019). Micro-structuration of a sol-gel architecture for channel waveguide / diffraction grating coupling. Opt. Mater..

[29] Demuth C., Varonier J., Jossen V., Eibl R., Eibl D. (2016). Novel probes for pH and dissolved oxygen measurements in cultivations from millilitre to benchtop scale. Appl. Microbiol. Biotechnol..

[30] Barczak M., McDonagh C., Wencel D. (2016). Micro- and nanostructured sol-gel based materials for optical chemical sensing. Microchim. Acta.

[31] Mills A., Graham A., O’Rourke C. (2014). A novel titania sol-gel derived film for luminescence-based oxygen sensing. Sens. Actuators B Chem..

[32] Briche S., Tebby Z., Riassetto D., Messaoud M., Gamet E., Pernot E., Roussel H., Dellea O., Jourlin Y., Langlet M. (2011). New insights in photo-patterned sol-gel derived TiO2 films. J. Mater. Sci..

[33] Tien P.K., Ulrich R. (1970). Theory of the prism-film coupler and thin film light guides. J. Opt. Soc. Am..

[34] Morand A., Kaur Y., Gri M., Ardila G., Benech P. (2019). Glass integrated optic waveguides combining optical grade dicing and ion-exchanged planar waveguide. Proc. SPIE OPTO.

[35] Kogelnik H., Ramaswamy V. (1974). Scaling rules for thin-film optical waveguides. Appl. Opt..

[36] Saleh B.E.A., Teich M.C. (2007). Fundamental of Photonics.

[37] Yariv A., Yeh P. (2006). Photonics: Optical Electronic in Modern Communications.

[38] Hugonin J.P., Lalanne P. (2005). Perfectly matched layers as nonlinear coordinate transforms: A generalized formalization. J. Opt. Soc. Am. A.

[39] Bucci D., Martin B., Morand A. (2012). Application of the three-dimensional aperiodic Fourier modal method using arc elements in curvilinear coordinates. J. Opt. Soc. Am. A.

[40] Lakowicz J.R. (1999). Principles of Fluorescence Spectroscopy.

